# Dental Anesthesia

**Published:** 1919-12

**Authors:** A. F. Smith


### DENTAL ANESTHESIA

BY DR. A. F. SMITH.

No single anesthetic agent, regardless of its character, is sufficient to produce and maintain anesthesia in all cases, but through the careful selection of an anesthetic agent, following a most thorough physical examination, the selection of the anesthetic should be made. The anesthetist, viewing this important branch of medicine and dentistry from this angle, is certain to achieve greater results and his cases of mortality will be much lower or wanting than the anaesthetist who administers one anesthetic agent to all patients regardless of their phychic and physical condition.

We must admire the anesthetist who is broad enough to familiarize himself with all anesthetic agents, and one who can see the good which can be accomplished through the medium of each of them and also realizes the value of a combination of pain relieving agents. It is not possible to secure 100 per cent. success with any single anesthetic.

Conductive anesthesia and nitrous oxide are both necessary in order to obtain the best results. If I were to make a distinction between the two methods, I would make it as follows: Nerve blocking the most valuable for the operative dentist, and nitrous oxide-oxygen most valuable for the man whose operations extend beyond those of operative dentistry. It is indeed hard to draw the line between these two important methods, and I see no reason for so doing, and I can only repeat that both methods should be thoroughly mastered and then employ the method indicated.

Anesthesia is a distinct science and the evolution which it has undergone during the past few years is gratifying, and this one link is one of the strongest in the chain of medical and dental education. We should look at this wonderful advancement with pride, not only because of the fact that the relieving of pain for our fellowmen is a great blessing but because most of the credit of advancement in scientific research in both general and local anesthesia goes to the dental profession of America. The goal of medicine and dentistry as practiced today is efficient results backed by thoroughness accomplished through the medium of clinical research.

I have been very careful to observe and to inquire of many men as to what extent they employ nerve blocking. I have found that many operators are laboring under the impression they are employing the method of nerve blocking, and in reality they are not. Local anesthesia has a wide and extensive classification. It is divided up into many divisions and subdivisions, and the mere injecting of some local anesthetic solution does not mean nerve blocking by any means. Nerve blocking is defined as that method of producing anesthesia in a circumscribed area by the injecting of the anesthetizing solution in or near the nerve trunk at a distant point from the field of operation; or, it can be defined as that method of anesthesia produced following the injection of the solution near the nerve trunk at any point between the periphery and the brain. This is truly nerve blocking and is only one of the great branches of local anesthesia. The dentist who states he is employing nerve blocking and is merely injecting the solution into the peridental membrane or gum tissue is wrong.

It is not possible to discuss the different methods of local anesthesia nor that of nitrous oxide-oxygen anesthesia but we must stick to the broad subject of anesthesia. The essayist spoke of methods practiced years ago, and I desire to compliment him upon the stand he has taken. The old methods were good in their day and answered their purpose for those who mastered them. We are aware of the fact it was not possible to obtain as efficient results with the older methods which were somewhat crude as compared with results obtained from the efficient methods at our disposal today for those who master them. We are living in an age of progress and the dentist of today who does not grasp the tool of efficiency and familiarize himself with those agents and methods which the sciences of medicine and chemistry offer, is not keeping abreast with progress. I think you will agree with me when I make the statement that when we view the broad field of dentistry that we have few experts who are capable of administering nitrous oxide and oxygen for an indefinite period of time to the patient possessing some pathological condition. This not only holds true for prolonged cases but for the case of short duration many times. To give a patient nitrous oxide-oxygen and administer it scientifically are two different things. It is self-evident that when the anesthesia is of short duration, which is generally the case in dental practice, the element of danger does not enter into the situation so gravely as when it is administered over a long period of time. Any layman with a few moments instruction could hold the anesthetic mask on the patient and produce asphyxia accompanied by cyanosis, the so-called anesthesia. There is a great difference between asphyxia and anesthesia. This layman could not administer nitrous oxide-oxygen for a prolonged period of time, but it requires someone experienced and capable who understands the signs and symptoms of anesthesia and the dangers, and the one who thoroughly understands the therapeutiaction of the compound nitrous oxid and the element oxygen.

The above statement with reference to nitrous oxide-oxygen not only holds good with that method but with nerve blocking as well. The anesthetist who inserts the needle into the deep tissues, blocking the various nerve trunks including the extraoral and intraoral methods, must thoroughly understand what he is doing. He must understand the true meaning of asepsis and possess a thorough knowledge of anatomy of the parts he is injecting, the physiologic and toxic action of the agent employed must also be understood and a thorough knowledge of the technic of nerve blocking injection. Unless these points are mastered, I can see the handwriting on the wall. One of the most efficient methods yet presented to our profession will be discarded by many in the same way as nitrous oxide-oxygen has been discarded by those operators who did not thoroughly understand its administration.

The time has passed when anyone can say with any degree of conscience, "Oh! anyone can administer an anesthetic." We will admit that almost anyone can pour ether or chloroform on a mask or hold the nitrous oxide inhaler and produce narcosis. We will also admit than anyone can cut off a leg or anyone can attempt to fill a root-canal, but there is a striking difference between the results of poor technic and good technic. We must remember there is something more in the practice of dentistry than merely operative procedures, and we must remember there is something more besides mortality and accidents in producing anesthesia—there is the patient's mental suffering previous to the operation. The many doubts, misgivings, and the fear of our patient are often caused more by the thought of the anesthetic that of the operation. A little confidence in the man behind the mask or the man holding the hypodermic syringe adds greatly to the comfort of the patient and the maintenance of an even anesthesia if a general anesthetic is employed, or the correct depth and direction of the needle if nerve blocking is employed.

When we stop to consider this subject of anesthesia from the patient's standpoint, it is a matter of grave importance. Place yourself in your patient's position and I doubt not that you will understand and appreciate more fully the cause of a rapid pulse, quick breathing and an anxious face. These factors plus mental anguish are manifest on the approach of the stranger who it to destroy thought, feeling and strength by giving you something of which you know little or nothing. I dare say after selecting your surgeon or your dentist for an operation requiring an anesthetic, be it one of the deep nerve blocking injections or the administration of a general anesthetic, your greatest concern will be, who shall administer the anesthetic.

It has been my good fortune, but not my pleasure, to observe in the different hospital clinics many patients nearly drowned with ether and smothered with gas, in the hands of those who knew little about what they were doing. I have seen ether poured on the mask until it was dripping from the patient's face, the mask being over the patient's face, the patient coughing and resisting and the anesthetist requesting them to breathe easily. I have also observed patients rendered cyanotic by giving nitrous oxide. Ninety per cent. of the graduates in medicine today have had little or no actual training in the scientific administration of anesthetics and with few lectures upon this important subject. Anesthesia is a distinct science. It demands careful consideration and study. All details must be mastered. It will no doubt be of interest for you to know that there was a great need for experienced anesthetists in the war. The field of anesthesia, scientifically speaking, is of comprehensive magnitude, and the dentist should by no means look upon it as a mere side line of dentistry and medicine, and those who master this subject will find that it is of immeasurable value and a source of pronounced appreciation and gratification. Science has given both medicine and dentistry countless new ideas during the past few years, but I am quite sure none of them are appreciated on the part of the patient so much as that of the relief of pain. It is wonderfully satisfying to observe the pronounced advancement in the science of anesthesia, and it is especially gratifying to see the vast number of our profession taking hold of the opportunities which science offers us today. Anesthesia is of vital importance to our profession, and we should recognize the importance of properly applied anesthetics and the careless administration of both general and local anesthetics should be discontinued and relegated to the rear of progressive dentistry. Carelessness in the administration of anesthetics is usually due to the practitioner having only limited knowledge of the physiologic and toxic effects of the agents employed.—*Journal N. D. A.*, July, page 632.

